# Association of Sleeve Gastrectomy With the Management of Ovarian Hyperstimulation Syndrome: A Retrospective Study

**DOI:** 10.7759/cureus.102456

**Published:** 2026-01-28

**Authors:** Asem Alfagih, Basheer Alkudumani, Zeyad Abualiat, Abdulrahman Alfadhel, Ali H Alsharedah, Tahir Mubarak

**Affiliations:** 1 Department of Reproductive Medicine and Infertility, Women’s Health Specialist Hospital, King Abdulaziz Medical City, Riyadh, SAU; 2 Department of Obstetrics and Gynaecology, Women’s Health Specialist Hospital, King Abdulaziz Medical City, Riyadh, SAU

**Keywords:** gastric sleeve, gastric sleeve surgery, length of hospital stay (los), ohss, ovarian hyperstimulation syndrome (ohss), sleeve gastrectomy

## Abstract

Introduction: The rise in global obesity is a major concern with particular implications for female reproductive health. Obesity is considered a risk factor for menstrual disturbances, female infertility, and reduced success rate of assisted reproductive technologies (ARTs). Weight reduction represents a promising therapeutic approach for addressing infertility in female patients who are obese. Nowadays, bariatric surgery is regarded as the most efficacious long-term intervention for morbid obesity, leading to enhanced reproductive health. The impact of bariatric surgery in terms of association and management has also been sparsely studied, and evidence regarding OHSS management after bariatric surgery is limited, highlighting a literature gap.

Objective: Primarily, this study aimed to investigate the association of sleeve gastrectomy with patients who developed ovarian hyperstimulation syndrome (OHSS) in terms of length of hospital stay.

Results: Interestingly, sleeve gastrectomy patients had longer hospital stays, averaging 5.3 ± 0.5 days, as compared to 3.3 ± 2.1 days among non-sleeve gastrectomy patients (p = 0.026).

Conclusion: The association of sleeve gastrectomy with the management of OHSS manifested as a longer hospital stay. We hypothesize that the reduced oral fluid tolerance after the sleeve gastrectomy may contribute to the increased length of hospital stay.

## Introduction

The World Health Organization (WHO) defines obesity as a "chronic complex disease associated with abnormal accumulation of fat that might impair health. Obesity is clinically defined by a body mass index (BMI) of 30 kg/m² or greater" [1]. Over the past years, there has been a global rise in obesity. Epidemiological data reveal a near-tripling of obesity prevalence from the period of 1975, and it is projected to increase to affect 18% of adults, which is equal to 1.02 billion [2]. Obesity is more common in females than in males. If this rising trend continues, by 2025, the global obesity prevalence will reach 21% in females and 18% in males [3]. Obesity has a wide spectrum of complications that can be categorized as metabolic, anatomic, and physiologic. Metabolic complications include type 2 diabetes, hypercholesterolemia, steatotic liver disease, chronic kidney disease, and atherosclerotic cardiovascular disease [4, 5]. Osteoarthritis, gastroesophageal reflux disease, and obstructive sleep apnea are examples of anatomical complications associated with obesity [4, 5]. Psychological complications include stigmatization, which will lead to social and financial discrimination [6]. Obesity affects female reproductive health in various ways. Obesity is considered a risk factor for menstrual disturbances, female infertility, and reduced success rate of assisted reproductive technologies (ARTs) [7, 8]. Obesity impairs female fertility in multiple processes, including hormone dysregulation, metabolic changes, and chronic low-grade inflammation, which all interfere with the normal reproductive function [9]. Weight reduction represents a promising therapeutic approach for addressing infertility in obese females, with lifestyle modification, pharmacological, and surgical approaches representing this strategy. While lifestyle modifications and increased physical activity might benefit some obese females, the majority of them fail to achieve considerable weight reduction by this means [9]. This issue led to the intense interest in bariatric surgery as a treatment modality for obesity-related infertility. Bariatric surgery is now widely regarded as the most effective long-term treatment for morbid obesity, resulting in improved reproductive health. [10]. A recent systematic review further reported that bariatric surgery is associated with improvement in the fertility outcomes, including higher cumulative live birth rates after ART [11]. Ovarian hyperstimulation syndrome (OHSS) is a feared complication with an incidence rate of 30% of in vitro fertilization (IVF) cycles [12]. OHSS develops due to an increased vascular permeability of the capillaries of the ovaries and peritoneum under the influence of ovarian vascular endothelial growth factor hypersecretion, which will lead to a shift of fluid from the intravascular to the extravascular compartment and significant ovarian enlargement [13]. OHSS is classified into four forms: mild, moderate, severe, and critical. The mild form is the most prevalent [14]. The most common reported risk factors for developing OHSS include young age, low body weight, history of OHSS, profound hyperstimulation protocols, high number of preovulatory follicles, high serum E2, and the use of human chorionic gonadotropin (hCG) for final oocyte maturation [15]. The cornerstone of OHSS management is fluid resuscitation, electrolyte correction, and prophylactic anticoagulation to reduce the thromboembolic risk. In cases with significant ascites or hemodynamic instability, therapeutic paracentesis might be considered. The choice of outpatient versus inpatient management requires individualized clinical judgment, considering the disease severity, hemodynamic status of the patient, and the capacity for close monitoring [15]. Currently, there is no strong evidence linking bariatric surgeries with increased risk for developing OHSS. All proposed hypotheses are based on theoretical assumptions secondary to the hormonal response following the weight reduction. If OHSS develops in a patient following bariatric surgery, the restricted fluid intake poses a substantial obstacle in the management of OHSS; this particular entity has never been studied before, leaving a gap in the literature. This study aims to address this gap and specifically investigate the association of sleeve gastrectomy with the management of OHSS.

Literature review

Obesity causes infertility primarily through hormonal imbalances, which can lead to alterations in the hypothalamic-pituitary-ovarian (HPO) axis. Reproductive hormones, including gonadotropin-releasing hormone (GnRH), luteinizing hormone (LH), follicle-stimulating hormone (FSH), estrogen, and progesterone, are all regulated through the HPO axis [15]. The excess adipose tissue in women with obesity creates higher estrogen, which can lead to a disruption of the HPO axis. Higher estrogen inhibits the FSH, which is responsible for the formation and maturity of the ovarian follicles [16]. Another factor is insulin resistance, which is frequently noted in obese females and is considered one of the hallmarks of metabolic syndrome and OHSS [17]. Insulin and LH promote androgen synthesis in the ovary, resulting in a hyperandrogenic status where the circulatory levels of testosterone are high [18]. This hyperandrogenic status leads to follicular arrest and anovulation; additionally, insulin levels play a role in inhibiting the hepatic synthesis of sex hormone-binding globulin, which ultimately leads to higher levels of testosterone and anovulation [19]. Apart from the hormonal dysregulation, obesity causes chronic low-grade inflammation and consequently affects reproductive health. Adipose tissue is a source for inflammatory mediators, including interleukin-6 (IL-6), C-reactive protein (CRP), and tumor necrosis factor alpha (TNF-α) [20]. These inflammatory mediators are associated with a reduction in ovarian function, impaired follicular growth, poor-quality embryos, and altered endometrial receptivity. Additionally, the persistent chronic low-grade inflammation is associated with oxidative stress, which decreases the quality of the oocytes and increases the implantation failure rate [21].

The majority of females with obesity fail to achieve weight reduction using traditional methods. This led to an increased interest in bariatric surgery, which is regarded as the most effective long-term treatment for morbid obesity. Sleeve gastrectomy, Roux-en-Y gastric bypass, and adjustable gastric banding are types of bariatric surgeries showing improvements in the reproductive health of obese females [22]. In reproductive-age females, Roux-en-Y gastric bypass and sleeve gastrectomy are the most commonly performed surgeries [23]. Both surgeries led to substantial weight reduction, enhanced metabolic indicators, and the restoration of normal reproductive function [24]. While the Roux-en-Y gastric bypass is generally superior in terms of weight reduction and metabolic evidence, it carries a higher risk for malnutrition and fetal growth restriction [25]. Additionally, a significant reduction in the oral intake was noted after the bariatric surgery; this reduction was attributed to the reduced gastric volume and early satiety. In a recent study, the fluid reduction was reported to be 58% in Roux-en-Y gastric bypass and 49% in sleeve gastrectomy [26].

OHSS is an uncommon but serious complication associated with ART. OHSS is classified as mild, moderate, severe, and critical depending on the clinical presentation. The traditional description of the syndrome includes abdominal discomfort, abdominal distention, dyspnea, ovarian enlargement, ascites, electrolyte imbalance, hemoconcentration, and hypercoagulability [27]. The basis of the management of OHSS depends on fluid resuscitation, correction of the electrolyte imbalance, thromboprophylaxis, and fluid drainage in symptomatic ascites [27]. The current literature lacks guidance on the management of OHSS in patients post-bariatric surgeries. Fluid resuscitation and electrolyte balance are the cornerstones of the management protocols of OHSS. The applicability of these protocols in post-sleeve gastrectomy patients is limited due to the fluid restriction. This patient group has never been the subject of OHSS management research. The lack of evidence necessitates developing tailored OHSS management protocols that take into account the unique physiological status of post-sleeve gastrectomy patients, including restricted fluid intake and potential nutritional deficiencies. 

## Materials and methods

Study design

This study is a retrospective single-center observational design conducted in Women’s Health Specialist Hospital, King Abdulaziz Medical City in Riyadh, Saudi Arabia. Data were collected over a 10-year period from January 2014 to December 2024. The retrospective approach was selected to enable comprehensive case identification and minimize the recall bias by utilizing the data extraction from the electronic medical records (EMRs). 

Study population

The study population included women aged between 18 and 40 years who were admitted for the treatment of OHSS and were eligible for treatment at King Abdulaziz Medical City. All patients included in the study met the diagnostic criteria for OHSS set by the Royal College of Obstetricians and Gynaecologists (RCOG) guidelines [28]. Patients were divided into two groups: patients who had undergone sleeve gastrectomy at least 12 months prior to the ovarian stimulation cycle and a matched control group of patients who had not undergone bariatric procedures. 

Inclusion criteria

The inclusion criteria for this study were female patients aged 18-42 who were diagnosed with OHSS based on the RCOG guidelines [28] and required hospital admission for OHSS management. Patients were required to have complete EMRs available and be eligible for treatment in King Abdulaziz Medical City.

Exclusion criteria

The exclusion criteria included patients outside the determined age group and those who were managed on an outpatient basis. Additionally, patients were excluded if sleeve gastrectomy was performed in less than 12 months or if they had a history of bariatric surgery apart from sleeve gastrectomy. The study excluded patients with incomplete or missing EMRs.

Collection of the data

All the relevant data was collected through the EMRs. Collected variables included the demographic data, prior episodes of OHSS, timing of the OHSS onset (post-ovum pickup or post-embryo transfer (ET)), and ovulation triggering agents, including hCG or Decapeptyl. 

Study objective

Primarily, this study measured the duration of hospitalization, used as an indicator of clinical course and resource utilization. The length of the hospital stay was defined as the interval from the admission date to the discharge date, as documented in the EMRs. 

Ethical consideration

This study was conducted in accordance with institutional ethical standards. Subjects' privacy and confidentiality were assured. No identifiers were collected. All relevant data handling procedures complied with institutional guidelines for confidentiality and data protection. The Institutional Review Board (IRB) at King Abdullah International Medical Research Center (KAIMRC), Riyadh, Saudi Arabia, approved this study with ethical clearance NRR25/039/9, dated 29/10/2025. Permission to collect data from the hospital was obtained before commencing collection. 

Study hypothesis

We hypothesize that patients who have undergone gastric sleeve surgery require a longer duration of hospitalization; we also hypothesize that the reduced oral fluid tolerance after the sleeve gastrectomy may contribute to the increased length of hospital stay. This limitation could potentially influence the clinical management and recovery time compared to individuals without such a surgical history. If this hypothesis is validated, it would emphasize the imperative for increased vigilance during ovulation induction protocols in this patient demographic, especially in cases complicated by OHSS. Specifically, the increased likelihood of extended hospitalization in these patients may necessitate tailored management strategies to mitigate associated risks and optimize clinical outcomes.

Statistical analysis

The analysis of the data was done with IBM SPSS Statistics software, version 29.0.0 (IBM Corp., Armonk, NY, USA). The continuous variables were expressed as the mean (SD), median, interquartile range (IQR), and range. The categorical variables were presented as frequencies and percentages (N, %). The association between gastric sleeve status and the categorical outcomes (paracentesis, OHSS severity, readmission, and clinical features) was tested using chi-square. The differences in the continuous outcomes, such as hospital stay duration, were evaluated using an independent t-test. The multivariate logistic regression was performed to identify the independent predictors of the gastric sleeve association with OHSS outcomes. A p-value < 0.05 was considered statistically significant.

## Results

Sixty-five patients were included for the assessment of the impact of the sleeve gastrectomy on the management of OHSS. The majority of participants included in this study were between the ages of 26 and 30 years (25, 38.5%) and 31-35 years (20, 30.8%). These are followed by 36-40 years (14, 21.5%), while the smaller proportions were aged ≤25 years (three, 4.6%) and >40 years (three, 4.6%). Moreover, the mean age was 32.1 years (SD 4.7 years), with the median of 32 years and an IQR of 32-36 years, spanning 23 to 46 years overall. Regarding the bariatric status, most of the participants had no history of the gastric sleeve surgery (59, 90.8%), whereas there was only a small subset of included participants who underwent the procedure (six, 9.2%) (Table 1).

**Table 1:** Patient demographics and rate of Sleeve gastrectomy procedure (n=65) Data are presented as number (percentage) for categorical variables and as mean ± standard deviation (SD), median, interquartile range (IQR), and minimum to maximum for continuous variables. Age is reported in years. No inferential statistical testing was applied for this descriptive table.

Variable		Frequency N (%)
Age (years)	≤ 25 years	3 (4.6%)
	26-30 years	25 (38.5%)
	31-35 years	20 (30.8%)
	36-40 years	14 (21.5%)
	>40 years	3 (4.6%)
	Mean (SD)	32.1 (4.7)
	Median	32
	IQR	32-36
	Min-Max	23-46
Sleeve gastrectomy procedure	No	59 (90.8%)
	Yes	6 (9.2%)

Table 2 shows the clinical profile, management, and outcomes of the OHSS patients. Notably, most of the patients had no prior history of OHSS (60, 92.3%), while there were only a few participants who reported the occurrence of OHSS (three, 4.6%). The majority developed OHSS post oocyte pick-up (OPU; 53, 81.5%), compared to post ET (10, 15.4%), with two patients (3.1%) of unknown timing. The most frequent triggering agent was hCG (62, 95.4%), which is followed by Decapeptyl (one, 1.5%), and unknown in two cases (3.1%). Clinically, most were diagnosed with moderate OHSS (40, 61.5%), while 18 (27.7%) had severe, and five (7.7%) had mild disease. In terms of management, 18 patients (27.7%) required paracentesis, mostly once (15, 23.1%) and rarely twice (three, 4.6%). The median hospital stay was three days (IQR 3-4 days), with no cases of readmission.

**Table 2:** Clinical profile of ovarian hyperstimulation syndrome (OHSS) with management and outcome after sleeve gastrectomy (n=65) Data are presented as numbers (percentage) for categorical variables and as mean ± standard deviation (SD), median, interquartile range (IQR), and minimum to maximum for continuous variables. Hospital length of stay is reported in days. This table is descriptive, and no inferential statistical testing was performed.

Variables		Frequency N (%)
Ovarian hyperstimulation syndrome (OHSS)-related clinical profile		
History of OHSS	No	60 (92.3%)
	Yes	3 (4.6%)
	Unknown	2 (3.1%)
Timing of OHSS	Post embryo transfer (ET)	10 (15.4%)
	Post oocyte pick-up (OPU)	53 (81.5%)
	Unknown	2 (3.1%)
Triggering agent	Human chorionic gonadotropin (hCG)	62 (95.4%)
	Decapeptyl	1 (1.5%)
	Unknown	2 (3.1%)
Diagnosis	Mild OHSS	5 (7.7%)
	Moderate OHSS	40 (61.5%)
	Severe OHSS	18 (27.7%)
	Unknown	2 (3.1%)
Management and outcome		
Paracentesis	No	47 (72.3%)
	Yes	18 (27.7%)
	Once	15 (23.1%)
	Two times	3 (4.6%)
Hospital stay (days)	Mean (SD)	3.5 (2.1)
	Median	3
	IQR	3-4
	Min-Max	0-14
Re-admission	No	65 (100.0%)
	Yes	0 (0.0%)

**Figure 1:**
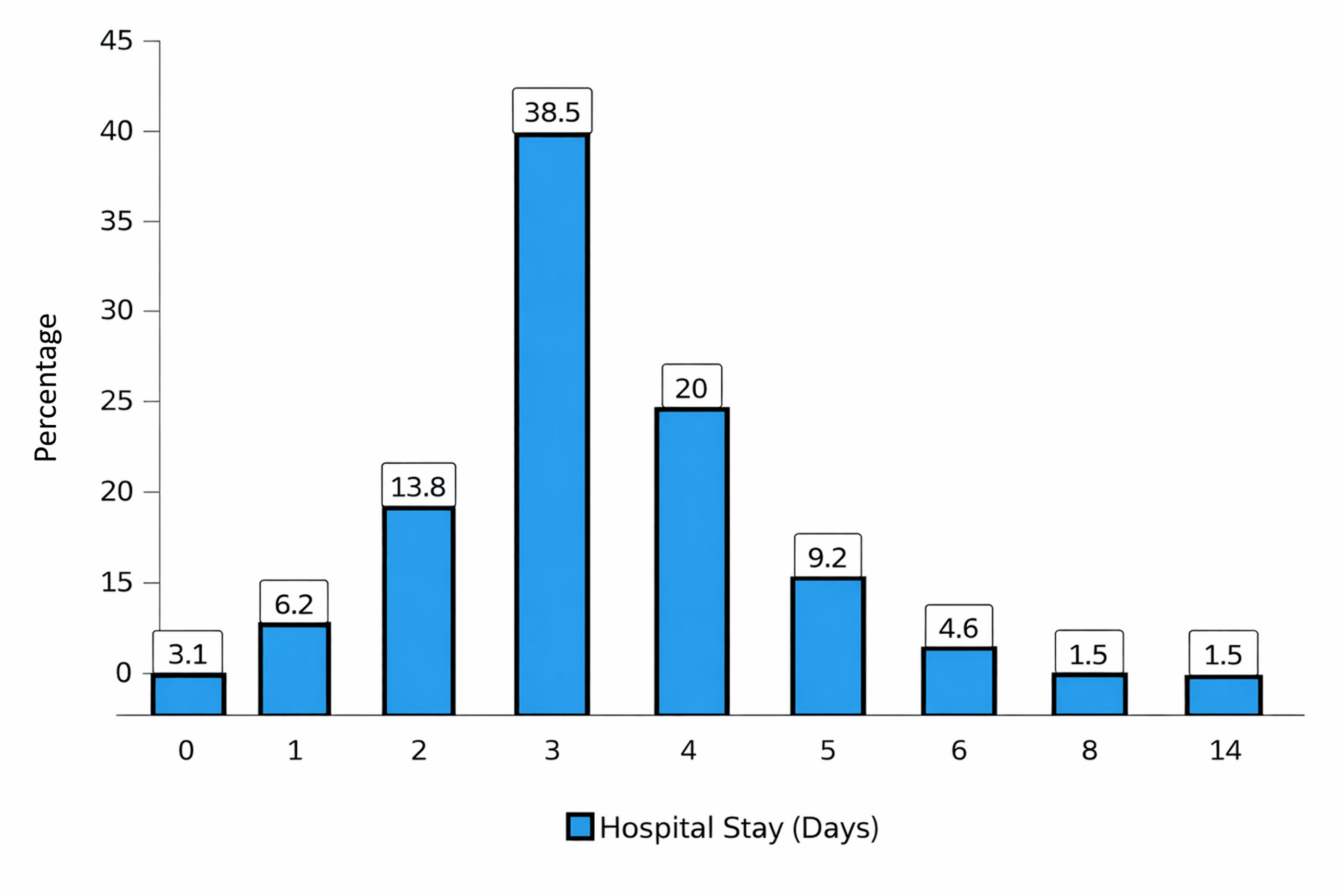
Distribution of length of hospital stay (n=65)

Figure 1 shows the distribution of the hospital stay among OHSS patients. Notably, most of the patients had relatively short admissions, with the majority staying three days (38.5%), which was followed by four days (20.0%) and two days (13.8%). The smaller proportions stayed five days (9.2%), one day (6.2%), or six days (4.6%). Moreover, very few patients had prolonged admissions, with eight, 10, and 14 days each accounting for 1.5%. Interestingly, the minority had no hospital stay at all (3.1%).

Table 3 explores the association between sleeve gastrectomy and OHSS outcomes. Notably, the patients with a sleeve gastrectomy history were less likely to require paracentesis, with only one out of six (5.6%) patients undergoing the procedure as compared to 17 out of 59 (94.4%) patients without a gastric sleeve (p = 0.526). Similarly, when the paracentesis was required, most sleeve gastrectomy patients had it only once (one, 6.7%), and none of them required two procedures, whereas three (100%) patients in the non-sleeve gastrectomy group underwent the two paracenteses (p = 0.645). There were no cases of readmission that occurred in either group (p = 1.000). Interestingly, sleeve gastrectomy patients had significantly longer hospital stays, averaging 5.3 ± 0.5 days, as compared to 3.3 ± 2.1 days among non-sleeve gastrectomy patients (p = 0.026).

**Table 3:** Association between gastric sleeve and outcome parameters of ovarian hyperstimulation syndrome Data are presented as number (percentage) for categorical variables and as mean ± standard deviation (SD) and median for continuous variables. Hospital length of stay is reported in days. Categorical variables were compared using the chi-square test, and continuous variables were compared using the independent samples t-test. A p-value < 0.05 was considered statistically significant.

Variables		Gastric Sleeve		Significant Values
		No N (%)	Yes N (%)	
Paracentesis	No	42 (89.4%)	5 (10.6%)	0.526 ^a^
	Yes	17 (94.4%)	1 (5.6%)	
Number of times paracentesis was performed	Once	14 (93.3%)	1 (6.7%)	0.645^ a^
	Two times	3 (100.0%)	0 (0.0%)	
Re-admission	No	59 (90.8%)	6 (9.2%)	1.000^ a^
	Yes	0 (0.0%)	0 (0.0%)	
Hospital stay (days)	Mean (SD)	3.3 (2.1)	5.3 (0.5)	0.026^ b^
	Median	3 Days	5 Days	

Table 4 shows the association between sleeve gastrectomy and clinical characteristics of OHSS. Sleeve gastrectomy was most common in the 36-40 years (three, 21.4%) group, which was followed by the ≤25 years (one, 33.3%) group, and there were no cases observed in the 31-35 years or >40 years groups, though the overall association was not statistically significant (p = 0.139). The prior history of OHSS was more frequent in the sleeve gastrectomy patients in terms of proportion (one, 33.3%) as compared with those without the sleeve gastrectomy (two, 66.7%), but the difference was not significant (p = 0.150). Similarly, OHSS following ET was slightly more common among sleeve gastrectomy patients (two, 20.0%) than non-sleeve patients (eight, 80.0%), while OHSS post-OPU was dominated by non-sleeve cases (49, 92.5%) (p = 0.219). The triggering with hCG was universal across groups, and diagnosis distribution (mild, moderate, severe) showed no significant differences (p > 0.05).

**Table 4:** Association of gastric sleeve procedure with age and clinical parameters of OHSS Data are presented as number (percentage). Age is reported in years. Comparisons between groups were performed using the chi-square test. A p-value < 0.05 was considered statistically significant. OHSS: ovarian hyperstimulation syndrome; ET: embryo transfer; OPU: oocyte pick-up; hCG: human chorionic gonadotropin

Variable		Gastric Sleeve Procedure		Significant Values
		No N (%)	Yes N (%)	
Age (years)	Up to 25 years	2 (66.7%)	1 (33.3%)	0.139^a^
	26–30 years	23 (92.0%)	2 (8.0%)	
	31–35 years	20 (100.0%)	0 (0.0%)	
	36–40 years	11 (78.6%)	3 (21.4%)	
	>40 years	3 (100.0%)	0 (0.0%)	
History of OHSS	No	55 (91.7%)	5 (8.3%)	0.150^ a^
	Yes	2 (66.7%)	1 (33.3%)	
Timing of OHSS	Post ET	8 (80.0%)	2 (20.0%)	0.219^ a^
	Post OPU	49 (92.5%)	4 (7.5%)	
Triggering agent	hCG	56 (90.3%)	6 (9.7%)	0.744^ a^
	Decapeptyl	1 (100.0%)	0 (0.0%)	
Diagnosis	Mild OHSS	5 (100.0%)	0 (0.0%)	0.745^ a^
	Moderate OHSS	36 (90.0%)	4 (10.0%)	
	Severe OHSS	16 (88.9%)	2 (11.1%)	

Table 5 shows the multivariate regression model for the assessment of the outcome predictors in relation to the gastric sleeve procedure. Notably, the paracentesis was not a significant predictor, with the patients undergoing the procedure showing an adjusted odds ratio (OR) of 0.290 (95% CI: 0.026-3.253, p = 0.315). The hospital stay demonstrated the trend toward significance, where each additional day increased the odds of the sleeve gastrectomy association by 1.416 times (95% CI: 0.984-2.039, p = 0.061), which suggested the potential relationship between the longer hospitalization and the gastric sleeve status. Notably, age did not emerge as a meaningful predictor, with an OR of 0.986 (95% CI: 0.824-1.181, p = 0.881), which indicated that there is no impact of the patient age on the sleeve gastrectomy outcomes.

**Table 5:** Multivariate regression model for sleeve gastrectomy, outcome, and age feature Results are presented as regression coefficient (B), standard error (SE), p-value, odds ratio (Exp[B]), and 95% confidence interval (CI). Hospital length of stay is reported in days, and age is reported in years. Multivariable logistic regression analysis was used. A p-value < 0.05 was considered statistically significant.

Variable	B	S.E.	Sig. Value	Exp(B)	95% CI for Exp(B)
Paracentesis (yes)	-1.239	1.234	.315	0.290	0.026 – 3.253
Hospital stay	0.348	0.186	.061	1.416	0.984 – 2.039
Age (years)	-0.014	0.092	.881	0.986	0.824 – 1.181

## Discussion

In the 21^st^ century, obesity is considered one of the global health challenges. The clinical definition of obesity is a BMI of 30 kg/m² or greater [1]. Over the past years, there has been a global rise in obesity. Epidemiological data reveal a near-tripling of obesity prevalence from the period of 1975, and it is projected to increase to affect 18% of adults, which is equal to 1.02 billion [2]. Obesity is more common in females than in males. If this rising trend continues, by 2025, the global obesity prevalence will reach 21% in females and 18% in males [3]. Obesity affects female reproductive health in various ways. Obesity is considered a risk factor for menstrual disturbances, female infertility, and reduced success rate of ART [7, 8]. Weight reduction represents a promising therapeutic approach for addressing infertility in obese females, with lifestyle modification, pharmacological, and surgical approaches representing this strategy. These developments led to the intense interest in bariatric surgery as a treatment modality for obesity-related infertility. Nowadays, bariatric surgery is considered the most long-term effective treatment for morbid obesity, resulting in improvement in reproductive health [10, 11]. 

The implications of the bariatric surgery in terms of the OHSS are not well-studied, highlighting a literature gap. The cornerstone of the OHSS management is fluid resuscitation and electrolyte imbalance. The physiological status post bariatric surgeries, more specifically the restricted fluid intake, poses a significant obstacle in the management of OHSS. This entity has never been studied before, highlighting a literature gap. 

This study primarily compares the length of hospital stay of patients who developed OHSS with a surgical history of gastric sleeve in comparison to patients who didn’t. We included all patients admitted for OHSS from 2014 to 2025. The sample size was 65 patients. Six patients had a history of sleeve gastrectomy, whereas the other 59 had no bariatric surgical history. Interestingly, the sleeve gastrectomy patients had significantly longer hospital stays, averaging 5.3 ± 0.5 days, as compared to 3.3 ± 2.1 days among non-sleeve patients (p = 0.026). The association of sleeve gastrectomy with the management of OHSS manifested as a longer length of hospital stay. We attributed this association to the restricted oral fluid intake post bariatric surgeries. This finding warrants extreme caution when dealing with patients post bariatric surgeries in terms of stimulation and triggering agents. A high index of clinical suspicion is mandatory throughout the cycle of ARTs for biochemical symptoms and ultrasound findings suggestive of OHSS. 

Despite the growing prevalence of bariatric surgeries among women of reproductive age, no evidence exists regarding their potential impact on the development and management of OHSS. This literature gap points to the need for well-structured studies to clarify the association between bariatric surgeries and OHSS, thereby yielding management protocols in this unique patient population.

The findings of this study should be interpreted considering the following limitations. The retrospective design of this study constrains the determination of causality between bariatric surgery and OHSS outcomes, as it primarily utilized medical records instead of prospective data collection with standardized protocols. Furthermore, the single-center study may limit the generalizability of the findings to other clinical settings or populations. Finally, the small sample size, especially in the bariatric surgery group, may have reduced the statistical power to identify significant differences between groups and increased the risk of type II errors.

## Conclusions

This study demonstrates that patients with a history of sleeve gastrectomy experience a longer hospital stay when presenting with OHSS in comparison to patients without such a surgical history. This finding warrants heightened vigilance in the management of this group of patients. Furthermore, this result highlights a notable gap in the literature, emphasizing the need to elucidate the underlying mechanisms and establish evidence-based management strategies tailored to this patient population.
